# Bioinformatics-Based Identification of CircRNA-MicroRNA-mRNA Network for Calcific Aortic Valve Disease

**DOI:** 10.1155/2023/8194338

**Published:** 2023-05-17

**Authors:** Linghong Song, Yubing Wang, Yufei Feng, Hao Peng, Chengyan Wang, Juncang Duan, Kejian Liu, Xihua Shen, Wenyi Gu, Yan Qi, Shan Jin, Lijuan Pang

**Affiliations:** ^1^NHC Key Laboratory of Prevention and Treatment of Central Asia High Incidence Diseases (First Affiliated Hospital, School of Medicine, Shihezi University), Department of Pathology and Key Laboratory for Xinjiang Endemic and Ethnic Diseases, Shihezi University School of Medicine, Shihezi, Xinjiang, China; ^2^Department of Cardiology, Jinhua Municipal Central Hospital, Jinhua, Zhejiang, China; ^3^Department of Cardiology, The First Affiliated Hospital, Shihezi University School of Medicine, Shihezi, Xinjiang, China; ^4^Australian Institute for Bioengineering and Nanotechnology, The University of Queensland, St Lucia, Australia; ^5^Department of Pathology, Central People's Hospital of Zhanjiang and Zhanjiang Central Hospital, Guangdong Medical University, Zhanjiang, Guangdong, China

## Abstract

**Background:**

Calcific aortic valve disease (CAVD) is the most common native valve disease. Valvular interstitial cell (VIC) osteogenic differentiation and valvular endothelial cell (VEC) dysfunction are key steps in CAVD progression. Circular RNA (circRNAs) is involved in regulating osteogenic differentiation with mesenchymal cells and is associated with multiple disease progression, but the function of circRNAs in CAVD remains unknown. Here, we aimed to investigate the effect and potential significance of circRNA-miRNA-mRNA networks in CAVD.

**Methods:**

Two mRNA datasets, one miRNA dataset, and one circRNA dataset of CAVD downloaded from GEO were used to identify DE-circRNAs, DE-miRNAs, and DE-mRNAs. Based on the online website prediction function, the common mRNAs (FmRNAs) for constructing circRNA-miRNA-mRNA networks were identified. GO and KEGG enrichment analyses were performed on FmRNAs. In addition, hub genes were identified by PPI networks. Based on the expression of each data set, the circRNA-miRNA-hub gene network was constructed by Cytoscape (version 3.6.1).

**Results:**

32 DE-circRNAs, 206 DE-miRNAs, and 2170 DE-mRNAs were identified. Fifty-nine FmRNAs were obtained by intersection. The KEGG pathway analysis of FmRNAs was enriched in pathways in cancer, JAK-STAT signaling pathway, cell cycle, and MAPK signaling pathway. Meanwhile, transcription, nucleolus, and protein homodimerization activity were significantly enriched in GO analysis. Eight hub genes were identified based on the PPI network. Three possible regulatory networks in CAVD disease were obtained based on the biological functions of circRNAs including: hsa_circ_0026817-hsa-miR-211-5p-CACNA1C, hsa_circ_0007215-hsa-miR-1252-5p-MECP2, and hsa_circ_0007215-hsa-miR-1343-3p- RBL1.

**Conclusion:**

The present bionformatics analysis suggests the functional effect for the circRNA-miRNA-mRNA network in CAVD pathogenesis and provides new targets for therapeutics.

## 1. Introduction

Calcific aortic valve disease (CAVD) is a condition caused by calcification of the aortic valve or aortic annulus, resulting in a hemodynamic manifestation of aortic valve stenosis or regurgitation [1]. It is a chronic progressive disease that increases in prevalence with age [2, 3], leading to an increasing proportion of acquired valvular heart disease. CAVD has become a significant factor of disease burden in the elderly [4], which has the characteristics of high morbidity and mortality [1]. Treatment of CAVD mainly relies on surgery [5], which includes surgical valve replacement and percutaneous valve prosthetic implantation. However, not all patients are eligible for surgical treatment. In terms of drug therapy, the antihypertensive drugs and statins are effective in treating atherosclerosis but do not reverse or slow the process of CAVD [6]. In conclusion, there is an urgent need to explore the key regulatory molecules in the pathogenesis of CAVD to provide new targets for its pharmacological treatment.

About 90% of the mammalian genome is transcribed into noncoding RNAs (ncRNAs), whose functions have not been fully studied [7]. With the development of deep RNA sequencing (RNA-seq) technology and novel bioinformatics methods, a wide variety of circular RNAs (circRNAs) types have been discovered and identified [8]. As a series of novel noncoding RNAs, circRNAs are characterized by a covalent closed loop structures lacking a 5′ cap or a 3′ Poly A tail [9]. The high abundance, relative stability, and evolutionary conservation of circRNAs distinguish it from traditional linear RNAs. CircRNAs has significant advantages in developing applications as a novel clinical diagnostic marker because it is able to better adsorb miRNAs from organisms than linear mRNAs and lncRNAs. However, the function of circRNAs is still unclear. There is growing evidence that belongs to competing endogenous RNAs (ceRNAs), which contain microRNA response elements (MREs). The specific RNAs with MREs can impair miRNA activity by sequestration, resulting in upregulation of miRNA target gene expression, which is known as ceRNA hypothesis [10]. The cirRNAs has been found to exert an important biological response in cardiovascular disease [11–13], but studies on CAVD are still limited. Wang et al. found that circRIC3, as a miR-204-5p sponge, positively regulates the expression of the calcification-promoting gene dipeptidyl peptidase-4 (DPP4), leading to CAVD [1]. Yu et al. reported that circRNA TGFBR2 positively regulates TWIST1 through sponge phagocytosis of miR-25-3p by inhibiting osteoblast differentiation and preventing valve calcification in human VICs [14]. These results reconfirm that circRNAs are critical in the development of CAVD. However, studies on circRNA-associated ceRNA networks in CAVD remain scarce. Therefore, studying of circRNA-miRNA-mRNA networks complements the lack of ncRNA in the exploration of CAVD pathogenesis. It is promising to find markers that can be used as diagnostic predictors of the disease and provide new insights for the treatment of CAVD.

**Figure 1:**
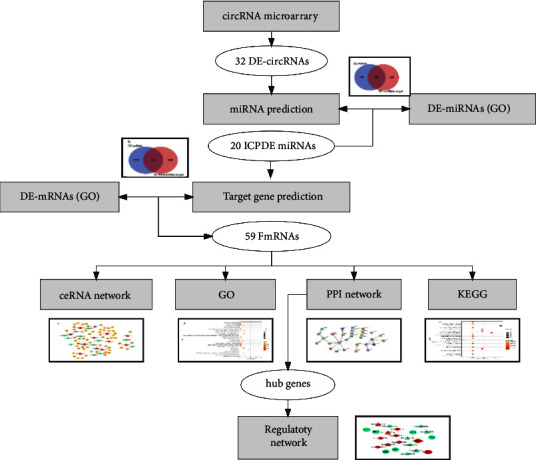
The schema of the bioinformatics analysis.

To investigate how the circRNA-miRNA-mRNA network regulates CAVD pathophysiological processes, we gain insight into the signaling regulation within the tissues leading to involvement in CAVD progression and discover relevant therapeutic targets. We screened CAVD-related circRNA, miRNA, and mRNA datasets in the Gene Expression Omnibus (GEO) database (Figure 1). By performing differential expression analysis, DE-circRNAs and DE-miRNAs targets were predicted. Following that, we constructed circRNA-miRNA-mRNA networks in CAVD. The common mRNAs (FmRNAs) were analyzed by gene ontology (GO) function enrichment analysis, Kyoto Encyclopedia of Genes and Genomes (KEGG) pathway analysis, and protein-protein interaction (PPI) network construction. We identified 8 hub genes from PPI for circRNA-miRNA-hub gene network visualization, and this regulatory network will be a potential therapeutic target for treating CAVD diseases. The schema of the bioinformatics analysis is shown in Figure 1.

## 2. Methods

### 2.1. RNA Array

The CAVD datasets from the National Center for Biotechnology Information (NCBI) GEO database (https://www.ncbi.nlm.nih.gov/geo/) were evaluated and screened. The GSE155119 dataset of circRNA expression was found in GPL26192 platform. The GSE87885 dataset of miRNA expression was found at GPL22555 platform. On the GPL10558 platform and GPL570 platform, we found the GSE83453 and GSE51472 datasets for mRNA expression. The calcified aortic valve contains 20 samples, including 3 circRNA samples, 2 miRNA samples, and 15 mRNA samples. The noncalcified aortic valve contains 19 samples, with 3 from the circRNA dataset, 3 from the miRNA dataset, and 13 from the mRNA dataset.

### 2.2. Screening for Differential Expression

Microarray datasets that provide RNA expression profile data in CAVD were imported into the R software and standardized with “impute” package [15–18]. The “LIMMA” package running in the R software analyzed the data for differential expression. |log Fold Change| > 2 and *p*-value <0.001 were considered to indicate significant differentially expressed circRNAs (DE-circRNAs). *p*-value <0.05 was considered to screen differentially expressed miRNAs (DE-miRNAs) and mRNAs (DE-mRNAs).

### 2.3. Prediction of DE-CircRNAs and DE-miRNAs Targets

The DE-circRNAs target miRNAs were predicted by using the online software, the Encyclopedia of RNA Interactomes (ENCORI). DE-miRNAs target genes were predicted by online websites, miRTarBase, TargetScan, and miRDB (Table 1), respectively. The mRNAs recognized by miRTarBase, TargetScan, and miRDB websites were considered candidate targets. The information of websites is shown in Table 1.

### 2.4. CircRNA-miRNA-mRNA Network

The common DE-miRNAs (ICPDEmiRNAs) were obtained from the intersection of DE-circRNA targets and the DE-miRNAs. Similarly, common DE-mRNAs (FmRNAs) were obtained by intersecting ICPDEmiRNAs targets with DE-mRNAs. Based on DE-circRNAs, ICPDEmiRNAs, and FmRNAs, we constructed circRNA-miRNA-mRNA network, which was visualized using Cytoscape software (version 3.6.1).

### 2.5. GO Function Analysis

The database for annotation, visualization, and integrated discovery (DAVID, https://david.ncifcrf.gov/) is an online web-based bioinformatics resource, which can provide tools for analyzing the function of large lists of genes/proteins [19]. The FmRNAs were input into DAVID online software to annotate the target genes with GO function. The results are visualized by R software.

### 2.6. KEGG Pathway Analysis

KEGG Orthology-Based Annotation System (KOBAS) is one of the most widely used web servers for gene/protein functional annotation and gene set enrichment. The KOBAS website was used to map FmRNAs to the KEGG pathway. KEGG pathway analysis visualization was performed using R software.

### 2.7. PPI Network

STRING (https://string-db.org/) database is the software for predicting protein-protein interactions. The PPI network of FmRNAs was established using the STRING database (version 11.0). A combined score of >0.4 was considered the cutoff to indicate a significant PPI pair.

### 2.8. CircRNA-miRNA-Hub Gene Network

Eight FmRNAs with a high degree of PPI were selected to find the relevant noncoding RNAs regulating them from the total DE-circRNAs and DE-miRNAs. These relevant data were imported into Cytoscape software (version 3.6.1) for analysis and visual graphing.

## 3. Results

### 3.1. Differential Expression of RNA Array

**Figure 2:**
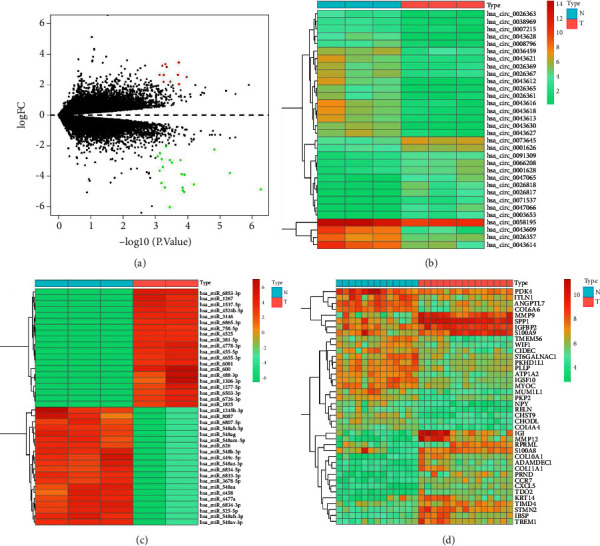
Differentially expressed RNAs. (a) Volcano plot of DE-circRNAs. Green represents down-regulation and red represents up-regulation. (b) Heatmap of 32 DE-circRNAs, *p*-value <0.001. (c) Heatmap of the DE-miRNAs with the most obvious up-regulation and down-regulation, *p*-value <0.05. (d) It is a heat map of DE-mRNAs, which is the most significant up-regulation and down-regulation, *p*-value <0.05. Darker colors indicate up-regulation, while lighter colors indicate down-regulation.

The “LIMMA” package is derived from R software, which allows analysis of differential expression in datasets. Thirty-two DE-circRNAs, 206 DE-miRNAs, and 2170 DE-mRNAs in the calcified aortic valve samples were compared with the uncalcified aortic valve samples. The DE-circRNAs are shown in Figures 2(a) and 2(b). DE-miRNAs (Figure 2(c)) and DE-mRNAs (Figure 2(d)) were visualized. For better display of the results, we selected the top 20 significantly upregulated and downregulated ones in order to show the statistically significant.

### 3.2. Construction of CircRNA-miRNA-mRNA Network

**Figure 3:**
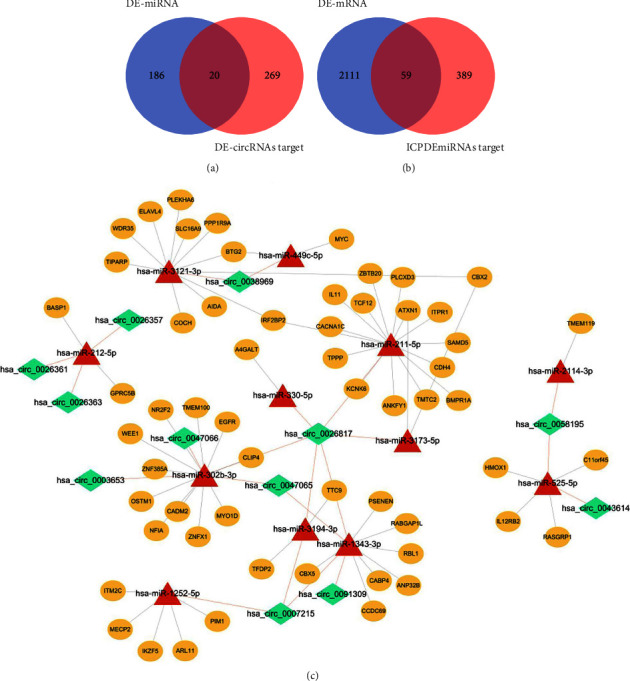
(a) Venn diagram of 206 DE-miRNAs and 289 DE-circRNAs targeting miRNAs. (b) Venn diagram of 474 target genes and 2170 DE-mRNAs predicted by ICPDEmiRNAs. (c) Construction of circRNA-miRNA-mRNA network. The diamond, triangle and oval shapes respectively circRNAs, miRNAs and mRNAs.

There were 289 DE-circRNAs targets miRNAs predicted by ENCORI. Twenty common miRNAs (ICPDEmiRNAs) were obtained by intersecting DE-miRNAs with DE-circRNAs targets (Figure 3(a)). These 20 ICPDEmiRNAs were subjected to target gene prediction in miRBase, TargetScan, and miRDB websites, and 474 target mRNAs were obtained. The 59 common mRNAs (FmRNAs) were the intersection of ICPDmiRNAs targets with DE-mRNAs (Figure 3(b)). Subsequently, the circRNA-miRNA-mRNA network was constructed, which contains 12 DE-circRNAs, 12 DE-miRNAs, and 59 DE-mRNAs (Figure 3(c)).

### 3.3. GO Function Analysis

**Figure 4:**
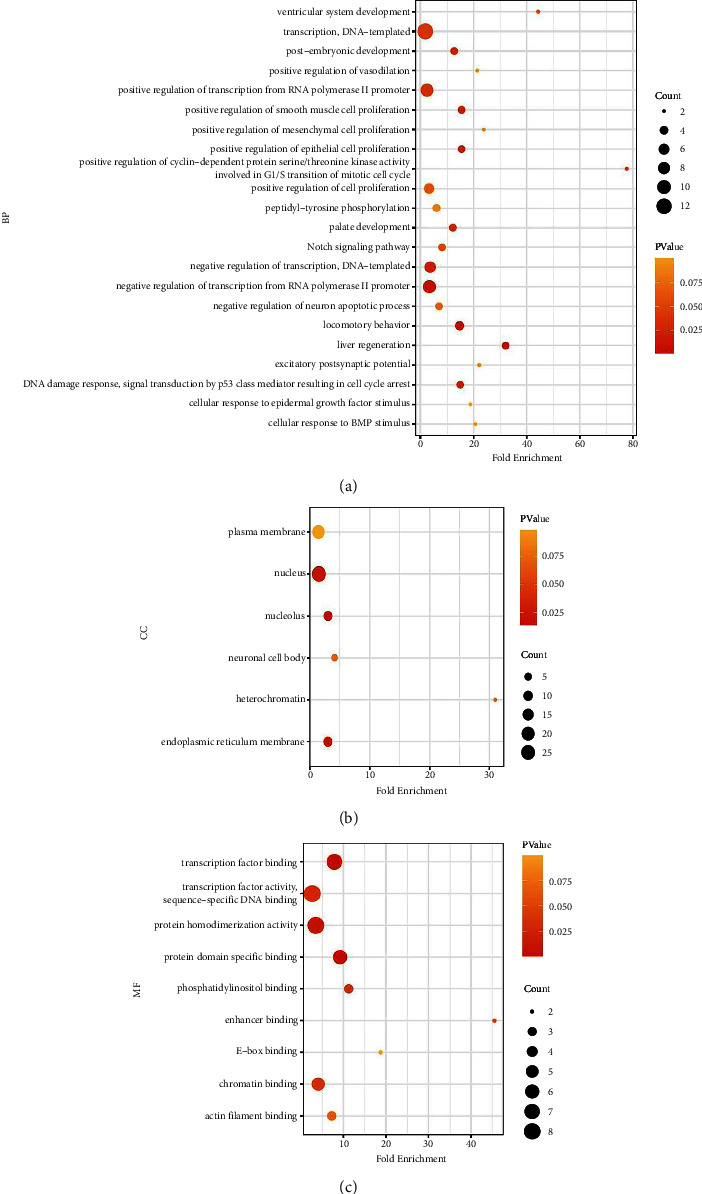
GO analysis for 59 FmRNAs. (a) Enrichment analysis on GO BP item. (b) Enrichment analysis on GO CC item. (c) Enrichment analysis on GO MF item.

GO functional annotation was used to analyze FmRNAs. Three GO categories were analyzed including biological processes (BP), molecular functions (MF), and cellular components (CC) (Figure 4). The 59 FmRNAs in the BP terms annotation function in GO mainly include transcription, DNA-templated, negative regulation of transcription from RNA polymerase II promoter, positive regulation of transcription from RNA polymerase II promoter, negative regulation of transcription, DNA-templated, positive regulation of cell proliferation (Figure 4(a)), nucleolus, endoplasmic reticulum membrane, nucleus, heterochromatin, neuronal cell body, and plasma membrane in the CC terms (Figure 4(b)). Protein homodimerization activity, transcription factor activity, sequence-specific DNA binding, transcription factor binding, protein domain specific binding, and chromatin binding pertained to the MF terms (Figure 4(c)).

### 3.4. KEGG Pathway Enrichment Analysis

**Figure 5:**
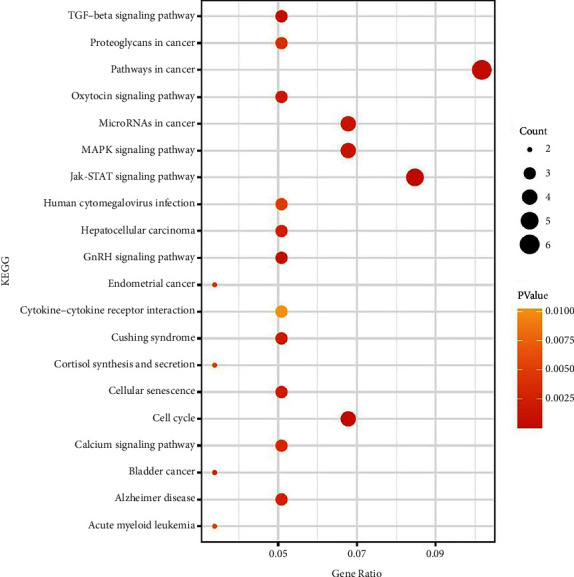
KEGG pathway analysis of FmRNAs. The larger the dot, the greater the degree and the richer the number of genes.

Understanding the enrichment pathways of these FmRNAs can gain further insight into the significance in CAVD. The KEGG pathway was used for enrichment analysis of these FmRNAs (Figure 5). FmRNAs are primarily involved in cancer pathway, JAK-STAT signaling pathway, and cell cycle and MAPK signaling pathway in KEGG pathway analysis.

### 3.5. PPI Network

**Figure 6:**
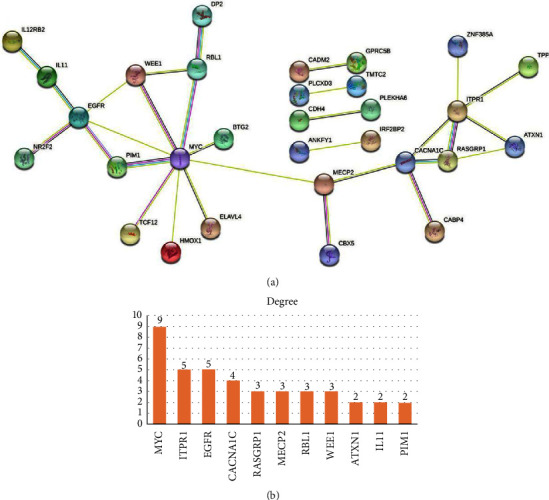
(a) FmRNAs PPI network. The protein corresponding to the FmRNAs is expressed through the circle, and the line represents the relationship between the protein and the protein. The more lines, the more important the protein. (b) Histogram of degree values in PPI networks.

**Figure 7:**
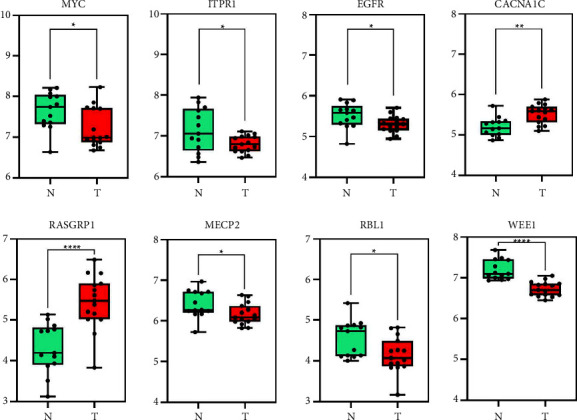
Eight hub genes expression in CAVD mRNA dataset. ^*∗*^*p* < 0.05, ^*∗∗*^*p* < 0.01, ^*∗∗∗∗*^*p* < 0.001.

Target gene data predicted by FmRNAs were uploaded to the STRING database for the construction of the PPI network (Figure 6). The MYC, ITPR1, EGFR, CACNA1C, RASGRP1, MECP2, RBL1, and WEE1 were the hub genes with high degree values. The hub genes expression was shown in Figure 7.

### 3.6. Construction of CircRNA-miRNA-Hub Gene Network

**Figure 8:**
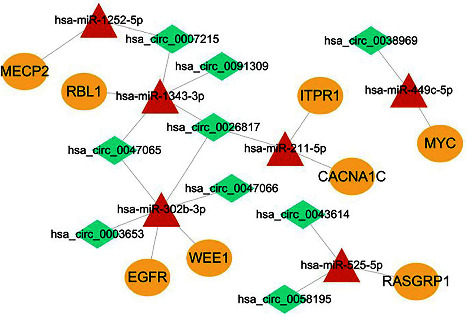
The circRNA-miRNA-hub gene regulatory network. The diamond, triangle and oval shapes respectively circRNAs, miRNAs and mRNAs. Green represents downregulated RNAs, red represents upregulated RNAs.

According to the PPI network, the 8 hub genes were obtained. In order to investigate the regulatory network, Cytoscape software (version 3.6.1) was used to construct and visualize these hub genes for the circRNA-miRNA-hub gene network (Figure 8). Based on the “miRNA sponge” function of circRNA, three groups of circRNA-miRNA-hub gene regulatory networks were identified including hsa_circ_0026817-hsa-miR-211-5p-CACNA1C, hsa_circ_0007215-hsa-miR-1252-5p-MECP2, and hsa_circ_0007215-hsa-miR-1343-3p-RBL1.

## 4. Discussion

CAVD, the most common valvular disorder, is the leading cause of aortic stenosis. The most effective treatment is surgery or interventional valve replacement [20], which has complications and does not guarantee long-term success [21]. There is an absence of approved pharmacological treatments to stop the progression or treat CAVD [22]. The ceRNA hypothesis has been proposed as a model for regulating gene expression during disease progression in recent years [10]. There is growing experimental evidence that multiple noncoding RNAs, including circRNAs, small non-coding RNAs, pseudogenes, and lncRNAs may have ceRNA activity [23]. More importantly, circRNAs, which are highly resistant to nucleases, maintain high abundance in the cytoplasm and better regulate miRNAs. However, it was only circRIC3 and circRNA TGFBR2 that were studied in CAVD disease [1, 14]. Construction of circRNA-miRNA-mRNA regulatory networks is essential to understand the pathophysiological progression of CAVD as the basis for developing novel therapeutics.

We have constructed circRNA-miRNA-mRNA regulatory networks based on the sponge activity of circRNA. Most circRNAs in the coexpression network remain unknown. Bioinformatic analysis of the DE-circRNAs target genes in network showed that transcription was the most important BP identified by GO analysis. Vadana et al. found that expression of SMAD and Runt transcription factors increased calcium deposition in CAVD [24]. The KEGG pathway of the target gene is significantly enriched in the cell cycle, MAPK and TGF-*β* pathway. It has been previously shown that MAP2K1 mutations activate p-ERK-dependent cell cycle progression and autophagy, exhibiting arterial valve stenosis [25]. Inhibiting the p38-MAPK signaling pathway can reduce ALP activity and calcification deposition to ameliorate aortic valve calcification [26].

We successfully established 3 circRNA-miRNA-hub gene networks relevant to CAVD, which include hsa_circ_0026817-hsa-miR-211-5p-CACNA1C, hsa_circ_0007215-hsa-miR-1343-3p-RBL1, and hsa_circ_0007215-hsa-miR-1252-5p-MECP2. Normal aortic valves are composed of valve endothelial cells (VECs) and valve interstitial cells (VICs), which play an important role in maintaining valve morphology and function [27]. Dysfunction of VICs and VECs is the key to the progression of CAVD. Upregulated hsa_circ_0026817 in CAVD may target hsa-miR-211-5p to regulate CACNA1C. It has been shown that miR-211-5p overexpression inhibits cell cycle by decreasing cyclin D1 levels [28]. Inhibition of cyclin D1 essentially abolishes fibrotic responses which are associated with VICs proliferation [29, 30]. Downregulation of miR-211-5p in CAVD leads to aortic valve fibrosis via the regulation of cyclin D1 in VICs. CACNA1C is the gene encoding the L-type voltage-gated Ca^2+^ channel [31]. The activation of cytosolic L-type Ca^2+^ channel leads to the entry of small amounts of Ca^2+^ into the cytoplasm and triggers Ca^2+^ release from the sarcoplasmic reticulum by activating ryanodine receptor 2 (RyR2). RyR2 was predominantly expressed in VICs, and inhibition of RyR2 prevents valvular calcification [32]. Matsui et al. identified high expression of CNCNA1C in calcified valves and verified the involvement of CACNA1C in CAVD progression by affecting valve calcification in VIC cells [33].

Down-regulated hsa_circ_0007215 in CAVD may regulate both hsa-miR-1343-3p/RBL1 and hsa-miR-1252-5p/MECP2. Upregulated miR-1343-3p in CAVD might directly influence valve endothelial cells (VECs) growth through the TGF-*β* signaling pathway. The surface of the heart valves is covered with VECs [34], which forms a barrier between the blood and the internal valve tissue [35]. In the aortic valve, TGF-*β*1 is predominantly localized to VEC and found to decrease the phosphorylation of RBL1 at the G1/S boundary, thereby inhibiting the development of cells into S phase [36, 37]. hsa-miR-1343-3p/RBL1 pathway was involved in CAVD by regulating the VECs cycle. Overexpression of miR-1252-5p might take part in CAVD by promoting MAPK signaling pathway [38], which has been shown to be involved in regulating Ca^2+^ entry into cells and mediating osteogenic differentiation of VIC in CAVD [39, 40]. MECP2, a target gene of miR-1252-5p, is an important regulator for the maintenance of normal cardiac development and myocardial structure [41]. The shorter e2 splice isoform of MECP2 can activate the MAPK pathway [42], which is involved in determining the structure of healthy heart [43]. Our findings suggest that 3 circRNA-miRNA-mRNA networks could be contributing factors for CAVD.

**Figure 9:**
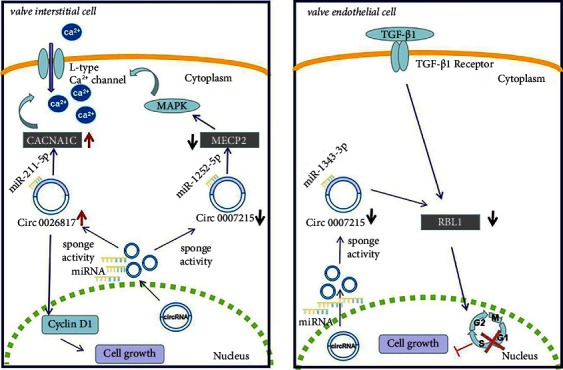
Schematic representation of the role of three circRNA-miRNA-mRNA regulatory networks in promoting CAVD. Has_circ_0026817-hsa-miR-211-5p-CACNA1C encodes enhanced L-type calcium channels that promote calcium influx into VICs, leading to calcification. In addition, hsa_circ_0026817 targets hsa-miR-211-5p through sponge activity, regulates cyclin D1, and promotes proliferation of VICs. Hsa_circ_0007215-hsa-miR-1252-5p-MECP2 regulates calcium channels through the MAPK pathway and is involved in calcification of VICs. Hsa_circ_0007215-hsa-miR-1343-3p-RBL1 is regulated by TGF-*β* signaling. RBL1 is inhibited in VEC, blocking G1-S phase cell cycle progression and regulating VECs proliferation. In hsa_circ_0007215-hsa-miR-1343-3p-RBL1, TGF-*β* signaling is able to regulate RBL1 to block cell cycle progression in G1-S phase and regulate proliferation of VECs.

In conclusion, the pathogenic effects of the ceRNA network in CAVD may be associated with the regulation of VICs and VECs. The identified 3 circRNA-miRNA-hub gene axes may constitute the underlying pathophysiology of CAVD (Figure 9). This offers new insights into pharmacological interventions for CAVD. In considering the multiple factors that are responsible for CAVD disease, including collagen accumulation and resident cytopathic remodeling [44], these circRNA-miRNA-mRNA axes could also be involved in CAVD formation. We addressed this issue through further analysis; hsa_circ_0026817-hsa-miR-211-5p-CACNA1C, hsa_circ_0007215-hsa-miR-1343-3p-RBL1, and hsa_circ_0007215-hsa-miR-1252-5p-MECP2 may be a new effective and potential target for the treatment of CAVD.

## 5. Conclusion

The establishment of CAVD is a result of the contribution of multiple regulatory factors. We constructed the circRNA-miRNA-mRNA regulatory network by microarray data mining and comprehensive bioinformatics analysis. It reveals that hsa_circ_0026817-hsa-miR-211-5p-CACNA1C, hsa_circ_0007215-hsa-miR-1252-5p-MECP2, and hsa_circ_0007215-hsa-miR-1343-3p-RBL1 axes may play a crucial part in CAVD and may provide new insights into the pathogenesis and therapeutic targeting of CAVD.

## Figures and Tables

**Table 1:** Websites for circRNA and miRNA targets prediction.

Name	Website
miRTarBase	https://mirtarbase.mbc.nctu.edu.tw/php/index.php
TargetScan	https://www.targetscan.org/vert/
miRDB	https://mirdb.org/
ENCORI	https://starbase.sysu.edu.cn/index.php
Bioinformatics &Evolutionary Genomics	https://bioinformatics.psb.ugent.be/webtools/Venn/

## Data Availability

The data used in this study are publicly available and allow unrestricted reuse through open licenses. All datasets in this study were downloaded from the GEO database. These datasets were taken from the following public domain resources: https://www.ncbi.nlm.nih.gov/geo/. The GEO public database allows researchers to download and analyze public datasets for scientific purposes.
